# Sustainable furniture rental that enhances the furniture experience of young migrants: A literature review

**DOI:** 10.1016/j.heliyon.2025.e41968

**Published:** 2025-01-16

**Authors:** Wei Liu, Siti Mastura Md Ishak, Mohd Faiz Yahaya

**Affiliations:** Faculty of Design and Architecture, Universiti Putra Malaysia, 43400, Serdang, Selangor, Malaysia

**Keywords:** Young migrants, Sustainable furniture, Green furniture, Furniture rental, Sustainable materials

## Abstract

Career transitions often lead young migrants to relocate, necessitating frequent disposal of furniture. Therefore, furniture rental offers a practical solution to this recurring issue. There is a need to establish sustainable furniture rentals to accommodate the unique needs of young migrants. The researchers used a ‘Literature Review Synthesis Process’ to analyse and integrate the collected information. The aim was to discover optimal solutions to address the challenges of furniture rental for young migrants, ensuring they no longer need to concern themselves with furniture disposal during relocation. The findings indicate that adopting sustainable materials is a strategy to fulfil the criteria of sustainability, cost-effectiveness, transportability, hygiene, dependability, aesthetic appeal, and damage recompense garners with significant approval.

## 1 Introduction

In recent decades, China's rapid urbanisation has led to rapid population growth in its large cities. As one of the fastest-growing cities, Shenzhen is favoured by migrants. According to data from the Shenzhen Municipal Bureau of Statistics, the city's permanent population (those who have resided in Shenzhen for more than half a year) increased by 2.6803 million people between 2016 and 2020. By the end of 2020, the permanent population of Shenzhen had reached 17.6338 million people [1]. Simultaneously, Shenzhen's high housing prices are a cause for concern for the city's residents. Data from the Shenzhen Bureau of Statistics illustrate that the average annual salary of urban workers (employees in state-owned, urban collective, joint-stock, and foreign-invested economies, Hong Kong, Macao, and Taiwan-invested economies, other economic units, and their affiliated institutions are managed and funded by them) in Shenzhen in 2020 was 139,436 yuan, whereas the average residential transition cost in Shenzhen for the same year was 52,808 yuan/m^2^ [1]. Therefore, most of the city's residents cannot afford the high housing prices and prefer to rent apartments.

Du (2019) pointed out that, in China, most urban migrants from Hong Kong and Beijing have experienced a change in residence within the past three years [2]. This situation has also arisen in Shenzhen. When people move homes, furniture disposal becomes a major challenge—they must pay extra for the transportation of furniture, and the furniture quality is often average, causing damage during transit and making it unusable thereafter [3]. Buying new furniture and disposing of the old ones are choices for some people. However, this leads to high resource consumption and waste generation [4]. Conversely, furniture leasing has gained recognition as a more sustainable method because it is cost-efficient and convenient and offers other advantages (e.g. freedom from burdens of ownership, novelty, and trialability) [5]. Therefore, young migrants are willing to use this option.

Unfortunately, the furniture rental services in China do not meet the needs of the migrants in Shenzhen. Therefore, it is essential to pay attention to the furniture needs of young migrants in Shenzhen during their rental period and integrate sustainable development goals to create suitable solutions for them. To achieve this, a sustainable furniture rental design strategy must be created to synthesise sustainability assessments, business models, and manufacturing [6].

This study adopted a ‘Literature Review Synthesis Process’ to analyse and integrate the collected information on sustainable furniture, furniture rental, and sustainable material applications. The aim was to identify the most suitable solutions to address the challenges of furniture rental for young migrants in Shenzhen.

## 2 Research Method

This study focused on furniture rentals and used the ‘Literature Review Synthesis Process’ [7] for developing raw writing materials. This literature review typology [[8], [9], [10]] contributes to understanding selected existing literature and identifying the appropriate theoretical background early in the research conception.

The furniture rental theme was divided into three sub-constructs: sustainable furniture, furniture rental, and sustainable material applications. The keywords were chosen based on sub-constructs such as young migrants, sustainable furniture, green furniture, furniture rental, and sustainable materials. Various relevant articles were searched using the Web of Science, Google Scholar, Scopus, and China National Knowledge Infrastructure. The top 174 most relevant articles were selected by conducting a title search and screening publications from 2015 to the present. Each article was assigned to the most relevant subtheme based on its relevance to the three subconstructs. Further review of scholars’ primary findings, how their findings support future research, and areas needing stronger emphasis in future research were identified. Finally, the 37 articles in total that were most likely to address the research question were retained (sustainable furniture: 12, furniture rental: 10, and sustainable material applications: 15).

The results of this study provide a comprehensive summary of each subconstruct. Subsequently, the prioritisation of cross-analysis, integration of possibilities, and comprehensive summaries are presented. Finally, the manuscript concludes by discussing potential integrated solutions for furniture rental to meet the needs of young migrants in Shenzhen.

## 3 Results

### 3.1 Sustainable furniture

This section reviews important articles considered valid by scholars from diverse backgrounds. This explains the related concepts of sustainable furniture, green furniture, and furniture recycling and reveals relevant content, such as consumer purchase intention for green furniture.

The importance of sustainable development has increased only over the past decade, making it a relevant and pressing issue [11]. A sustainable design is an important aspect of sustainable development. Sustainable design refers to the implementation of sustainable concepts through design practice, education, and research [12]. Sustainable design development is inseparable from green design, ecological design, product lifecycle design, and other concepts. Moreover, sustainable design requires integrating design behaviour into the ‘man-machine-environment’ system, which realises social value and protects natural value [12]. Thus, sustainable furniture is a specific design practice guided by the concept of sustainable design, which meets people's daily needs and the various requirements of sustainable development. Furthermore, practising sustainable design promotes the implementation of sustainable development goals.

Xu et al. (2021) highlighted the sustainable design concept in modern Finnish furniture. Their study emphasised that in sustainable furniture design, environmentally friendly materials (nontoxic, safe, renewable, and recyclable), green furniture packaging (recyclable packaging materials and technology that ensure no harm to the environment or people), and a simple structure should be selected to create a natural and original design for broader sustainable development systems [12]. Li and Zhao (2023) concurred with Xu et al. (2021) and pointed out the idea of building a sustainable development system that introduced the concept of sustainable development into the entire furniture lifecycle. Their study emphasised that the construction of a sustainable development system in the furniture industry should start with six specific requirements: sustainable design (sustainability of materials, shapes, structures, and workmanship), sustainable production (innovation in the use of biomass energy and innovation in intelligent technology), sustainable packaging (proper protection function, recyclable materials, reasonable structure, appropriate volume and capacity, reasonable transportation costs, and complete basic information), sustainable sales (intelligent, diversified, social, and mobile multiple media and technology applications), sustainable services (service forms such as trade-in, recycling, and furniture rental), and ‘zero-carbon’ emissions (upgrades the design plan; offsets the carbon footprint and carbon emissions to achieve zero carbon emissions) [13].

However, there are differences in design requirements between furniture provided for users to purchase and furniture exclusively for rental purposes. This is because rental furniture has higher resale, transportation, maintenance, and recycling requirements than traditional furniture. Xu et al. (2021) focused only on sustainable design research of furniture purchased by consumers. Similarly, Li and Zhao (2023) did not include rental furniture in their research.

Therefore, researchers concur with Xu et al.’s (2021) sustainable furniture design concept and Li and Zhao's (2023) sustainable furniture development system perspectives. This study examined sustainable furniture designs based on the needs of furniture rentals.

#### 3.1.1 Green furniture

Environmental, environmentally conscious, ecological, life-cycle, and sustainable designs are other terms used for green design. This is a new approach to industrial design that prioritises the creation of environmentally friendly goods, conservation of resources, and environmental protection [14]. Green furniture is a product that follows green design principles, supporting sustainable design development.

Xiong et al. (2020) highlight the current situation and manufacturing points of green furniture in China. Their study emphasised the concept of Green Furniture Manufacturing (GFM), which includes the following: green design (incorporating environmental factors and pollution prevention measures into the design during the design stage), green material selection (environmentally friendly materials), and green technology (based on traditional technology. Factors such as human health and safety, pollution prevention, and resource consumption throughout the product life cycle are integrated into comprehensive consideration. Furthermore, green packaging (packaging that minimises waste and conserves resources, does not pollute the environment, is simple to recycle or reuse, can break down organically, and supports sustainable development) and green recycling (recycling or reuse to reduce environmental contamination and enhance the use of resources) are considered [14]. Deng (2020) proposed a view similar to Xiong et al.’s (2020) furniture design based on green design concepts. Her study indicated that green furniture design should use environmentally friendly raw materials that can save natural resources while creating a healthier and more comfortable indoor environment. Moreover, she suggested that furniture should be freely assembled and disassembled to facilitate its use and recycling, reducing the cost of workforce, time, and capital. Additionally, green furniture should adopt green ecological manufacturing processes to reduce the consumption of raw materials for furniture production, minimising the waste generated during the production process and protecting the environment [15].

Specifically, Zhu and Niu's (2022) research on green materials highlighted their requirements in office furniture. Their study emphasised that the selection of desk structural materials, decorative materials, and auxiliary materials (e.g. connectors) should be combined with the economic benefits to the enterprise. Comprehensively considering aspects such as service life, recyclability, and reusability will achieve the dual goal of enterprise development and environmental protection. Furthermore, desk packaging materials should be repurposed and recycled to preserve resources and the environment [16].

Nevertheless, as with research on sustainable furniture, because rental furniture has more requirements for resale, transportation, maintenance, and recycling than ordinary furniture, there are differences in their design requirements. Although Xiong et al. (2020) and Deng (2020) have similar views, their study focused on furniture for consumers. Moreover, their research did not include the characteristics of rental furniture. Research on green materials has not combined Zhu and Niu's (2022) studies with the characteristics of rental furniture.

Therefore, researchers agree with Xiong et al.’s (2020) and Deng's (2020) green furniture concepts. This study is based on Zhu and Niu's (2022) study and combines the concept of green furniture with research on rental furniture.

#### 3.1.2 Furniture recycling

Resource shortages and waste have severely restricted the sustainable development of the furniture industry. There is an urgent need to solve this problem [17]. When furniture items reach the end of their lifecycle, not recycling them will result in resource waste and environmental pollution. Based on statistical data, China generates six billion tons of municipal waste every year, of which various types of waste wood products account for approximately 1%—equivalent to 60 million tons or 85 million m3 of waste. Therefore, furniture recycling can solve the challenge of numerous abandoned furniture items and waste resources and reduce the strain on the environment. Additionally, it will contribute to realising the concept of a circular economy and sustainable development [14].

David et al.’s (2019) study recommended the proper disposal of discarded products using recycling concepts. Their study emphasised that the government should formulate strict regulations to allow producers and users to coordinate and promote product recycling. Specifically, producers' recycling responsibilities must be expanded first to make them responsible for recycling the products they produce. Second, consumers have the responsibility to return products they no longer use to producers and help them recycle and reuse them, profiting from them together [18]. Additionally, in the research on China, Xiong et al. (2022) agreed with David et al.’s (2019), which pointed out the current situation of second-hand furniture recycling in China and analysed the problems existing in recycling used furniture from the perspectives of the government, furniture manufacturers, and consumers (imperfect systems, high costs, and weak awareness). In response to these obstacles, they emphasised corresponding solutions for the recycling of used furniture in China. In other words, the government should establish a waste furniture recycling network and supervise and support furniture production enterprises to assume recycling responsibilities. The next solution is for government agencies to formulate recycling standards and improve their systems. Additionally, furniture manufacturers must improve the durability of furniture, delay furniture replacement, design furniture easy to recycle, improve the recyclability of furniture, and develop new models and technologies for furniture recycling. Meanwhile, consumers must improve their consumption concepts, care about environmental education, and reduce social pressure to increase the efficiency of recycling old furniture [17].

Further, Yang and Zhu's (2021) research on value-added issues after recycling highlights the recycling and value-added design ideas for used wooden furniture. Their study emphasised four principles that must be considered in value-added design following the recycling of used furniture: monomer and system (converting a single reuse problem into a systematic product design problem based on waste substrates), whole and detail (considering various factors comprehensively), function and emotion (functional and emotional integration) [20], process technology, and design concept (combining technology and design). Moreover, they emphasised seven factors that influence value-added design: consumer groups, modelling, colour, material, structure, culture, and scope (expanding the recycling scope). Combining the requirements of these principles and factors can effectively promote recycling and value-added issues of wooden furniture [19].

However, they did not combine David et al.’s (2019)and Xiong et al.’s (2022) concepts with furniture rental. Yang and Zhu's (2021) study was conducted only on wooden furniture, and other sustainable materials could be developed based on their theory.

Therefore, the researchers agree with David et al. (2019) and Xiong et al. (2022). This study is based on Yang and Zhu's (2021), emphasising the synergy between companies and users. We focused on the value-added issue of rental furniture recycling with other sustainable materials.

#### 3.1.3 Consumer purchase intention

Recognising how customers evaluate design concepts and their effects during the buying process can assist furniture manufacturers in determining the critical elements they should emphasise in attracting customers and meeting their needs. Furthermore, by researching the significance of environmental accountability on consumer behaviour, it is possible to obtain insights into the economic convenience of companies investing in environmental tactics, including eco-design practices, to increase their market share and competitiveness [21]. The results support the implementation of sustainable design.

Xu et al. (2020) identified the determinants of consumers' willingness to purchase green furniture. They emphasised that perceived behavioural control (the controllability to perform a certain behaviour), physical health concerns (a conscious attitude towards the health of your family and yourself), and experience (consumers who have purchased green products) significantly and positively affect consumers' willingness to purchase green furniture. Moreover, research illustrates that environmental awareness has an indirect effect on purchase intention through perceived behavioural control [22]. Barbaritano and Savelli (2021) focused on environmental issues and investigated how consumers' environmental responsibilities affect their purchase intentions for furniture. Their study highlighted that design can serve as a three-dimensional structure based on functional, aesthetic, and symbolisms (i.e. the iconic and communicative meaning of a product). While functional and aesthetic factors influence consumers’ evaluation of a design [23], the symbolism of the design is the main factor influencing purchase intention. Additionally, concern for environmental issues moderates the relationship between the symbolism dimension of design and purchase intention—if customers care about environmental issues, they tend to be more affected by the symbolism dimension of design, increasing their purchase intention [21].

However, there are differences between leasing and purchasing in terms of consumption form. Leasing refers to the acquisition of the right to use a product rather than the ownership of the product. There may be differences in consumer willingness to rent and their willingness to purchase furniture. Although Xu et al. (2020) and Barbaritano and Savelli (2021) failed to pay attention to consumers' consumption intentions for furniture rental, the researchers agree that their research results have a certain correlation with research on users’ intentions for furniture rental. Therefore, this study combines consumer attitudes with sustainable research on rental furniture.

#### 3.1.4 Symbolic attributes

Consumers’ purchasing intentions are mainly influenced by the symbolic dimensions of the design [21]. Therefore, creating symbolic attributes is essential.

The use of sustainable materials (especially wood and wood materials, which people are familiar with) is significant in creating symbolic attributes. Xu et al. (2021) pointed out that, in Finland, many designers advocate the concept of natural home furnishing. This results in Finnish furniture exhibiting the characteristics of clear wood grains and rough and simple lines, which makes people feel a natural, quiet, and simple atmosphere. For example, in Finland, many sofas, tea tables, and base cabinets are made of raw wooden materials that retain traces of the branches' natural growth. Furthermore, rattan furniture is comfortable and breathable, has a fresh appearance, and smells natural, making people feel refreshed and cool. This reflects the Finnish designers’ pursuit of wood [12]. Today, Finnish furniture is popular in the global market, demonstrating its success in creating symbolic attributes, such as eco-friendly, simple, and healthy, through the use of natural and sustainable materials.

Guzel (2020) agrees with Xu et al. (2021), who pointed out survey results on consumer preferences and usage attitudes towards wood, wooden products, and furniture. The participants in this study stressed that wood can be defined as a material that brings pleasure and happiness to people and relaxes them mentally and emotionally. Nevertheless, as natural wood is an expensive material, consumers consider using alternative materials such as wood composites (e.g. particleboard). The participants stated that the benefits of these materials included low production costs and a variety of colours and designs. Furthermore, the study observed that products with the word ‘environmentally friendly’ on the label and those that cause less environmental harm during the production process help increase consumers' willingness to buy them. Designing and producing environmentally friendly wood products are important for consumers [24].

Han et al. (2022) agreed with Guzel (2020) and Xu et al. (2021) who conducted a survey on public awareness of and preferences for wood culture. The survey results demonstrate that 79 % of the general respondents and all expert respondents agree that the use of wood will be crucial in solving climate and environmental problems and bring beneficial health and emotional effects. Moreover, wood is considered a friendlier traditional building material than reinforced concrete or stone. The intention to use wood in architecture and interior design was as high as 86.6 %, with a particular emphasis on its superior quality. Wooden furniture, wooden buildings, and their use as indoor/outdoor materials are examples of avenues where wood is actively utilised [25].

Although Xu et al. (2021) only focused on Finland, Guzel (2020) studied Kayseri, and Turkey. Han et al. (2022) conducted research in South Korea. Differences among users in various countries and regions were evident. Furthermore, other sustainable materials can contribute to the creation of symbolic attributes. Therefore, this study aims to explore the potential of sustainable materials based on the characteristics of users in Shenzhen to create symbolic attributes favoured by users of rental furniture.

In conclusion, the researchers agreed with the concept of sustainable design of green furniture and the view of a sustainable development system for furniture. Based on consumer attitudes, this study combines the design of green sustainable furniture that meets the requirements of furniture rental, focusing on the recycling and value-addition of sustainable materials and the symbolic attributes created by sustainable materials. Additionally, it emphasises the synergy between companies and users to develop feasible sustainable rental furniture.

### 3.2 Furniture rental

This section reviews important articles considered valid by scholars from diverse backgrounds. This explains consumers’ attitudes towards leasing businesses and leasing furniture. Further, it explains the related concepts of furniture leasing.

The rental business model supports sustainability. Leasing can maximise the use of the same product and reduce product waste while meeting more user needs. This business model has attracted increasing attention among young people. Du (2019) indicated that most urban migrants live in rented houses. They described their current living situation as a ‘makeshift arrangement’. In this case, they did not want to spend too much energy or money on housing. Although many urban migrants complained about the poor quantity and quality of the existing furniture, they did not consider purchasing new furniture, unless necessary. This is because many landlords prohibit tenants from leaving their belongings when they leave, making the process of transporting and disposing purchased furniture highly cumbersome [2]. In this context, furniture rentals provide opportunities for development as a business model that meets the needs of urban migrants.

#### 3.2.1 Users’ willingness to rental business

User participation plays a pivotal role in the leasing business, directly influencing its future development based on user willingness to engage in leasing activities. Understanding users' willingness to rent businesses will help understand consumers’ collaborative consumption motivations from a broader perspective.

Kim and Jin (2020) identified consumers' motivations for collaborative consumption. Their study emphasised five basic dimensions of collaborative consumption of consumer goods: concern for sustainability (consumers who are environmentally aware and reduce social waste display a stronger tendency to engage in collaborative consumption), social collaboration (consumers who desire to be part of a larger shared community are often attracted to collaborative consumption practices), cost-saving (collaborative consumption may appeal to price-sensitive consumers who are looking for ways to save money on goods), variety-seeking (consumers choose collaborative consumption methods to obtain rich product choices), and fun (consumers can derive hedonic value by searching for and finding unique items that can be physically accessed or owned) [26]. Fota et al. (2019) and Kim and Jin (2020) have similar views and highlight the factors driving users' willingness to participate in the leasing business. Their study highlighted five positive drivers: sustainability (positive impact on the environment and maximum product use), economic efficiency (saving cost and time, and obtaining more resources), trust (level of trust in the rental company and the products and services it provides), security (products and services provided to users with less uncertainty and risk), and knowledge of the rental business (previous understanding and familiarity with leasing business knowledge). Furthermore, complexity (the knowledge structure related to leasing is cumbersome, and the learning cost is high) is highlighted as a reverse driver of users’ willingness to participate in the leasing business [27].

Nonetheless, as in previous studies, there were differences in population characteristics across regions. Kim and Jin (2020) failed to focus on consumer groups in Shenzhen and did not use furniture rental as a more detailed research object. Additionally, Fota et al.’s (2019) study concentrated on Germany and the research content did not involve furniture.

Therefore, the researchers agree with the five basic dimensions of collaborative consumption of consumer goods proposed by Kim and Jin (2020) and combined it with Fota et al.’s (2019) driving factors of users' willingness to participate in the rental business. Furthermore, they focused on the attitudes towards the rental business among young migrants in Shenzhen while exploring the factors that influence their willingness to rent furniture.

#### 3.2.2 Users’ attitude and willingness towards furniture rental

Furniture rentals are a subdivision of the leasing business. Understanding users' willingness to rent furniture will help us understand the specific content of furniture rental that meets consumer needs.

Du (2019) highlighted design research on lifestyles related to space and furniture consumption by urban migrants in Hong Kong and Beijing. The scholar emphasises that sufficient services should be considered from the perspective of functional categories, styles, grades (level of value perceived by consumers), prices, and accessories (decorative accessories, home appliances, etc.) to build a feasible furniture rental model. Further, scholars have emphasised that migrants from Hong Kong and Beijing have different priorities when renting furniture. Hong Kong focuses on practical functions and the full utilisation of space, whereas Beijing pays more attention to improving living quality and taste [2]. Contrary to the research population in Du's (2019) study, Lu (2021) pointed out the consumer's attitude towards furniture rental in Shanghai. The scholar emphasised that the most critical driving factors for furniture rental among consumers were ‘meeting temporary needs’, ‘being able to try different furniture styles and brands’, and ‘reducing the worry of moving’. Moreover, barriers to consumers renting furniture include hygiene issues—furniture may be soiled or have pests and bugs from previous users. Furthermore, furniture suppliers may not fully disinfect them. Additionally, some respondents said they would not accept second-hand products because ‘they have been used by other users’, ‘damage compensation’, and ‘low trust in the furniture rental business model’ [4].

Research on German users by Buch et al. (2021) emphasised trend orientation (pursuit of the trend) and perceived risk (uncertainty about the possible negative consequences of using a product or service) as the most important factors influencing consumers' furniture rental intentions. In addition, sustainability value (sustainable impact on the economy, society, and environment), economic value (cognitive trade-offs between a product's perceived benefits and specific costs), and materialism (material possession preference) significantly influence consumer willingness to rent furniture [3].

However, as with the problems faced by previous research, Du's (2019) study focused on consumers in Hong Kong and Beijing and Lu's (2021) on consumers in Shanghai. Buch et al. (2021) focused only on German consumers. The demographic characteristics, user attitudes, and willingness of these groups are somewhat different from those of young migrants in Shenzhen.

Therefore, the researchers agree with the point of view proposed in Du (2019) about gaining user favour based on styles and prices. Additionally, it needs to meet the requirements of ‘meeting temporary needs’, ‘trying different furniture styles’, ‘reducing the worry of moving’, ‘hygiene issues’, and ‘damage compensation’. Furthermore, the perceived risk, sustainable value, and economic value that affect consumer willingness to rent furniture have been recognised by researchers [3].

#### 3.2.3 Furniture rental service

Furniture rental companies provide rentals. Research on furniture rental companies and the rental services they provide can help better integrate the needs of consumers with the development of rental companies, achieving the development of furniture rental.

Kongehl and Sander (2021) indicated how fashion and furniture retailers can incorporate leasing into their business models and overcome consumer resistance. Their study emphasised the need to meet the needs of environmentally conscious and trend-oriented consumers by providing added environmental value and a convenient, flexible rental experience. Additionally, retailers must implement fair usage-based pricing, quality, and cleanliness checks and ensure the durability of rental products to overcome consumer resistance [11].

Schoonover et al. (2021) pointed out the obstacles that home-furnishing companies face in implementing a rental business model. Their study emphasised the barriers faced by the rental model regarding financial and economic concerns (cash flow and financial risks), product design (satisfying different needs), capabilities (tracking products, product maintenance, warehousing, and distribution), relationships (cooperation between manufacturers, retailers, and third parties), end users (unfamiliar with the concept of rental products, differences in cost expectations, ease of use, variety of services, and hygiene issues), and policy (taxes and institutional support) [28]. Interestingly, Schoonover et al. (2022) pointed out the corporate framework of furniture rental based on their previous study—Schoonover et al. (2021), which emphasised that furniture rental companies resonate with consumers by emphasising financial benefits, freedom from the burdens of ownership (owned products cannot meet the changing needs of consumers), novelty, convenience, and sustainability as secondary roles. Moreover, companies prefer to rely on intangible ideas, such as aspirations, self-expression, and homeliness (creating a home), to convey these concepts. Furthermore, companies gain traction with consumers by citing more specific content, such as the ownership burdens associated with transporting furniture [5].

This novel furniture rental subscription model differs from previous research. Soler (2020) has emphasised price (getting the right to use furniture at a lower price) and service (reliable delivery, assembly, and pickup) as the main drivers of furniture rental subscription models. Flexible leasing methods and sustainable concepts are conducive to the advancement of this model [29].

To adapt to the evolution of the internet era, users’ online consumption is a topic that most business models must address. Research on online furniture rentals can help improve furniture rental services.

Kapoor and Vij (2021) identified the attributes that lead to conversions on online furniture rental platforms. Their study emphasised six areas that can have a significant impact on online furniture rental conversions: perceived value (the trade-off between get versus pay), social satisfaction (gaining satisfaction by defining social status), customisation (meeting the needs of different users), psychological ownership (feeling proud of owning the product and developing a sense of attachment), career mobility (the process of changing profession, often associated with the location), and the last one is complementary services (free installation, disassembly, maintenance, delivery, and other services) [30].

However, demographic differences lead to differences in research results. Kongehl and Sander's (2021) study focused on Germany, Sweden, Switzerland, the Netherlands, and so on. Additionally, they only focused on the rental performance of traditional furniture products, ignoring the performance of new materials and forms of furniture rental. Similarly, Schoonover et al.’s (2021) study on Sweden focused on the company's perspective and user-related barriers based on what the company knows about its consumers. One advantage of this study was that it reflected actual consumer behaviour rather than attitudes or intentions. Conversely, research from the consumer perspective may identify different or additional barriers. In addition, they focused only on the rental company's promotional strategy for all consumers. However, Soler's (2020) study did not focus on consumer aspects, especially young users. Finally, the study by Kapoor and Vij (2021) was conducted in India. Although online rentals are growing rapidly, especially in urban areas, they are in their infancy. Furthermore, the sample size of the study remains limited, and the sampling strategy of the quantitative survey resulted in the underestimation of certain populations, limiting the generalisability of the findings.

In summary, this study is based on the characteristics of young migrants in Shenzhen, and explores a furniture rental model that is suitable for this population based on their willingness to rent furniture.

### 3.3 Sustainable material applications

This section explains some research findings on sustainable materials that can be used in sustainable furniture designs.

As the material basis of furniture, integrating sustainable materials into furniture leasing can better promote the development of furniture leasing and is a crucial step in achieving sustainable development. By 2025, the annual waste generated by humans is expected to reach 2.2 billion tons [18]. This has forced people to take measures to reduce waste generation. The use of sustainable materials can effectively mitigate waste.

The basic requirement of furniture products is that they be nontoxic, harmless, and pollution-free. Therefore, decomposable, renewable, and recyclable materials should be used as much as possible to produce furniture [12]. This coincides with the requirements for sustainable materials. For sustainable development, people should implement sustainable materials in furniture products, reducing waste generation and supporting sustainable furniture designs.

#### 3.3.1 Recycled aggregates and waste textiles

The construction industry is characterised by high resource consumption and large amounts of waste [31]. Concrete is widely used in the construction industry, and aggregates are the main constituent materials. Research on the sustainability of aggregates can help reduce waste and promote the use of sustainable materials.

In urban furniture research, Bedoya et al. (2019) emphasised the possibilities for sustainable concrete made from construction and demolition waste (CDW). Additionally, coal ash can be used in University Urban Furniture [32]. Sánchez-Roldán et al. (2020) agreed with Bedoya et al. (2019) that the use of recycled aggregates to produce concrete benches is technically feasible [31].

Textile waste increases as living standards improve. The use of textile scrap in wood-intensive furniture manufacturing is an acceptable option. Wang et al. (2023) proposed a sustainable furniture design based on recycling waste textiles. Their study emphasised how waste textile-starch composites could be used in sustainable furniture design to reduce the waste of natural resources. Waste textile-starch composites can effectively alleviate the difficulty of recycling waste furniture [33].

Additionally, it is known from the research of Bedoya et al. (2019) and Sánchez-Roldán et al. (2020) that sustainable concrete solves the waste problem and meets the strength requirements of furniture materials. However, the application of this material in rental furniture is questionable owing to the weight problem after the concrete is formed. In addition, Wang et al.’s (2023) study ignored the performance of rental furniture using waste textile-starch composites as materials.

In conclusion, alternative materials for furniture making that are suitable for rental purposes require several aspects such as manufacturability, physical appearance, material sustainability, and the feasibility of the design concept. Therefore, this study focused on the use of waste textile-starch composites as a sustainable solution that could lead to circular design.

#### 3.3.2 Recycled wood and wood materials

Traditionally, furniture has been made from wood and wood materials. Today, furniture is recycled, and other wood products are reused in the production of wood materials. Additionally, factories take care to utilise wood residue. Research on wood recycling can help reduce resource waste and environmental pollution, promote sustainable development, and reduce economic costs.

Pringle et al. (2018) reported a technically feasible method for transforming furniture wood waste into 3D printing materials for use in the furniture industry. They highlighted that the material was produced by mixing polylactic acid (PLA) particles with recycled wood waste. The material was tested in small quantities in the laboratory to verify its feasibility. Furthermore, they emphasised that as the weight percentage of wood furniture waste filler in a given batch of material increased, the properties of the 3D printed parts became closer to those of wood. When the weight percentage of wood furniture waste in the printed parts is at least 15 %, the parts begin to have the smell and texture of heavier wood, and emit a stronger odour. In addition, 3D printed parts can be sanded, stained, and painted like ordinary wood products, further increasing the value of these wood–polymer composite products. Throughout the project, discarded wooden parts or failed prints could be recycled and the materials could be reused [34].

Most studies in the waste wood sector have focused on wood-based boards. Among these, the largest number of studies have been on particleboards [35].

Particleboard is an engineered wood product created by hot-pressing wood chips together with synthetic resins or other appropriate adhesives at a specific pressure and temperature. Both global output and demand have increased recently. Particleboard is typically produced from wood chips sourced from forest thinning and wood waste. However, a reduction in the supply of natural forest wood makes it difficult to meet the growing demand for particleboard in the future. Consequently, using sustainable recycled materials in particleboard production is a feasible way to lessen the harm that the wood-based panel industry causes to the environment and increase resource efficiency [36].

Zamarian et al. (2017) studied the production of particleboard from waste furniture. They emphasised the relevant data obtained through experimental measurements, which demonstrated the potential of using waste furniture particles to produce particleboards [37]. Nguyen et al. (2023) agreed with Zamarian et al. (2017), who reviewed existing research on the production of wood-based boards using waste wood resources. The advantages of the technical and mechanical treatment processes for processing waste wood into particles were emphasised, indicating that particleboard is the first choice for the production of wood-based boards over fibreboard and oriented strand boards. Moreover, the core layer of the particleboard and oriented strand board products can be 100 % replaced by waste wood particles. Notably, the contaminants and low aspect ratio of recycled wood particles lead to a decrease in the physical and mechanical properties of board products. These disadvantages can be overcome by applying modern sorting technology to eliminate contaminants and appropriate shredding technology to increase the aspect ratio of the recycled wood particles. Additionally, the use of waste wood in the production of wood-based boards increases the risk of formaldehyde emissions from the particleboard and oriented strand board products. However, this risk can be addressed by using pre-treatment steps to reduce formaldehyde emissions (e.g. hydrothermal processes) or formaldehyde-free binders (e.g. polymeric methylene diphenyl diisocyanate) [35].

Abundant and renewable agricultural waste is an alternative raw material that can be used for particleboard production. Wood chips are the second largest cost element in particleboard production after resins, and these two elements account for more than 50 % of the total production costs. Therefore, replacing wood chips with lignocellulosic agricultural waste in particleboard production can significantly reduce production costs [36].

Kariuki et al. (2020) reported the use of agricultural crop residues as an alternative source of lignocellulosic materials in particleboard formulations, and various advances have been made to improve the properties of the resulting particleboards. This review presents the crop residues used as alternative lignocellulose material sources in particleboard formulations and the various advances that have been made to improve the properties of the resultant particleboards [38]. Lee et al. (2022) agreed with Kariuki et al. (2020), who reviewed and evaluated the current advanced technologies for particleboard production using various environmentally friendly agricultural biomasses, recycled wood waste, and by-products. They compared the performance of particleboards produced from different agricultural biomass materials (straw, stalk, bagasse, seeds/fruits, leaves, grass, and palm) in terms of mechanical parameters (particleboards made from bagasse performed the best). Further, they summarised the physical and mechanical properties of particleboards produced using recycled wood waste as a raw material. The results demonstrated that in many cases, particleboards made from alternative raw materials have similar or even better mechanical properties than conventional wood particleboards. Therefore, boards made of agricultural biomass and recycled wood waste can ensure sustainable development [36].

Nonetheless, Pringle et al. (2018) only focused on the application of recycled materials based on wood furniture waste in 3D printing additive manufacturing. Although additive manufacturing has the advantages of reduced waste and flexibility, considering factors such as cost control, batch-mechanised processing manufacturing methods remain popular among furniture manufacturers. Moreover, Zamarian et al. (2017) and Nguyen et al. (2023) only focused on the production of wood-based boards using waste wood resources (including waste furniture) and failed to pay attention to the performance of these boards in rental furniture. Similarly, Kariuki et al. (2020) and Lee et al. (2022) only focused on particleboards made from alternative raw materials and failed to pay attention to the performance of these particleboards in rental furniture.

Therefore, based on Pringle et al. (2018), researchers should focus on the performance of materials recycled from wood furniture waste in mass-produced rental furniture. Based on Zamarian et al. (2017), Nguyen et al. (2023), and Lee et al. (2022), researchers should focus on the performance of boards made of recycled wood waste and agricultural biomass materials in rental furniture.

#### 3.3.3 Recycled paper

A group of scholars is more interested in applying recycled paper materials than sustainable concrete and sustainable waste textiles.

Paper is used daily in many forms such as newspapers and office supplies. Furthermore, it is used in education, commerce, and packaging. The production of paper has a huge impact on the environment. Research demonstrates that increasing paper recycling by 50 % and 75 % reduces the annual consumption of 2,171,750 and 3,257,625 trees, respectively. Paper recycling, as an environmentally friendly solution, is becoming increasingly important for the sustainable development of the paper industry [39].

The paper and pulp industries frequently employ recycled paper as a raw material. It is a suitable material to replace wood in the production of fibreboards [40]. Some scholars have focused on the application of recycled paper as a sustainable construction material. Bahrami and Jafari (2020) pointed out the environmental benefits and impact of recycled paper as a sustainable material for landscape architecture. Their study emphasised that recycled paper can be obtained from the daily consumption of paper towels, drawing and faxing paper, cloth rolls, and so on. As it is inexpensive, lightweight, prefabricated, and recyclable, it can be used as a sustainable and creative material in construction [39]. Venkatesan et al. (2023) pointed out the possibility of using cardboard as a building material. Their study emphasised that cardboard, which overcomes certain limitations (such as water absorbency and fire resistance), can be used as a substitute for wood to provide improved environmental benefits [41].

Research on furniture by Jaramillo et al. (2018) indicated that recycled cardboard tubes were sustainable materials that can be used in street furniture. Their study emphasised the experimental and design results of recycled cardboard tubes to demonstrate their potential as materials for sustainable furniture [42]. Similarly, Sobotková et al. (2019) used recycled paper pulp to design furniture that adheres to sustainable design principles. Their study emphasised the fabrication and testing of paperboards made from recycled pulp to demonstrate the potential of paper pulpboards as tabletop materials [43]. Subsequently, Sobotková et al. (2021), based on their previous research—Sobotková et al. (2019)—pointed out that producing paper substrates from recycled post-consumer paper for furniture design was a feasible option. Their study highlighted that paper-polylactic acid (paper-PLA) boards are an alternative to wood-based items (e.g. desktops) made from post-consumer materials such as cardboard (CB) and office paper (OP). These boards can be recycled to create new boards that meet the sustainable and circular manufacturing requirements. Additionally, a comparison of the physical and mechanical properties of cardboard (CB) and office paper (OP) demonstrated that the former performed better [40].

However, the studies in Bahrami and Jafari (2020) and Venkatesan et al. (2023) focused only on the construction field. In addition, the studies by Jaramillo et al. (2018), Sobotková et al. (2019), and Sobotková et al. (2021) did not focus on the performance of their materials in rental furniture.

In conclusion, this study focused on the use of recycled paper as a rental furniture material because it is easy to obtain from daily wastepaper. Furthermore, it is a feasible alternative owing to its low cost, light weight, prefabricated recyclability, and good mechanical properties.

#### 3.3.4 Corrugated paper

Corrugated paper is primarily used for packaging products [44]. With the development of the e-commerce economy, the express delivery business volume has surged, leading to an increase in the use of corrugated paper [45]. Therefore, research on the sustainable application of corrugated paper materials helps improve research on recycled paper materials and supports the realisation of sustainable development goals.

Sales et al. (2018) conducted research on double-layer corrugated cardboard as an alternative material to environment-friendly furniture. Their study emphasised that corrugated cardboard can be used in furniture design and that corrugated cardboard is a material with good processability [44]. In addition, Liu and Zhang (2021), pointed out that the design of paper furniture was based on the secondary use of express corrugated boxes. Their study emphasised the need to pay attention to the characteristics of corrugated cardboard when using express corrugated boxes to design paper furniture, and to avoid overly complex shapes that make the structure fragile and reduce its strength. The corrugated cardboard furniture components were selected based on their functionality and load-bearing requirements. Simultaneously, the corrugated box itself has creases, and the treatment of the original creases must be considered during redesign [45].

However, Sales et al.’s (2018) study did not focus on the performance of corrugated paper in rental furniture. Meanwhile, Liu and Zhang (2021) focused only on the direct redesign of express corrugated boxes rather than prefabricated corrugated cardboard. Although this study has a certain degree of novelty, it also has certain limitations in the design of corrugated furniture owing to the size, shape, and degree of damage to the corrugated boxes.

In summary, this study found that the characteristics of corrugated paper need to be further explored. As this study examines the suitability of sustainable materials to make diverse, flexible, and practical rental furniture, the focus will be on the application performance of sustainable materials such as recycled paper and other materials with similar mechanical properties (e.g. waste textile-starch composites, agricultural biomass and recycled wood waste) in the design process.

#### 3.3.5 Reliability of sustainable boards

To intuitively demonstrate the performance of boards made of sustainable materials, we conducted a literature survey and extracted 24 sets of research data from high-quality literature published from 2015 to the present (2024) for relevant statistics. The statistical analysis included the mechanical and physical properties of the boards. The results are summarised in Table 1.

**Table 1:** Performance characteristics of sustainable boards made from selected materials.

ID	Target composite	Adhesive	THK (mm)	MOE	MOR	IB	SWR	TS	WA	D	REF
				(N/mm^2^)	(N/mm^2^)	(N/mm^2^)	(N)	(%)	(%)	(kg/m^3^)	
1	Recycled paper boards	Urea-formaldehyde	7	3019.93	14.29	0.41	Face:	24 h:52.93		1078.67	[43]
	(Deinked pulp)						542.95				
	Boards										
2	Sugarcane bagasse-citric acid particleboards	Citric acid	9	3361	17.38	0.73	Face:	24 h:5.32	24 h:42.92	800	[46]
		(15 %)					220				
3	Sugarcane bagasse-citric acid particleboards	Citric acid	9	3429	18.22	0.98	Face:	24 h:5.18	24 h:30.47	800	[46]
		(20 %)					292				
4	Sugarcane bagasse-citric acid particleboards	Citric acid	9	3944	21.88	1.03	Face:	24 h:4.43	24 h:29.48	800	[46]
		(25 %)					393				
5	Standard recycled wood particleboards	Urea-formaldehyde	12	2097	10.40			24 h:40.0	24 h:101.5	651	[47]
6	Recycled wood particleboards (Damaged furniture and pallets, crates, baskets, packages)	Urea-formaldehyde & bark	12	2189	10.60			24 h:29.1	24 h:86.1	646	[47]
7	Recycled wood particleboards (Damaged furniture and pallets, crates, baskets, packages)	Urea-formaldehyde & beech sawdust	12	2104	10.70			24 h:27.9	24 h:84.5	652	[47]
8	Recycled wood particleboards (Damaged furniture and pallets, crates, baskets, packages)	Urea-formaldehyde & pine sawdust	12	2247	10.60			24 h:28.6	24 h:83.9	649	[47]
9	Recycled wood particleboards (Damaged furniture and pallets, crates, baskets, packages)	Urea-formaldehyde & wood powder	12	1645	8.00			24 h:29.7	24 h:86.0	643	[47]
10	Discarded furniture particleboards (Particles of furniture discards10 %+particles of Pinus 90 %)	Urea-formaldehyde	13	1956	13.10	0.96	Face:	2 h:4.82	2 h:13.09 24 h:34.85	692	[37]
							1185	24 h:14.19			
							Top:				
							945				
11	Discarded furniture particleboards (Particles of furniture discards 25 %+particles of Pinus 75 %)	Urea-formaldehyde	13	1804	12.21	0.93	Face:	2 h:5.54	2 h:16.02 24 h:40.47	697	[37]
							1033	24 h:14.78			
							Top:				
							1034				
12	Discarded furniture particleboards(Particles of furniture discards 50 %+particles of Pinus 50 %)	Urea-formaldehyde	13	1689	11.97	0.72	Face:	2 h:5.99	2 h:12.34 24 h:42.09	703	[37]
							1148	24 h:15.98			
							Top:				
							1127				
13	Discarded furniture particleboards(Particles of furniture discards 75 %+particles of Pinus 25 %)	Urea-formaldehyde	13	1452	10.17	0.66	Face:	2 h:7.34	2 h:19.28 24 h:42.68	713	[37]
							1093	24 h:15.60			
							Top:				
							982				
14	Discarded furniture particleboards(Particles of furniture discards 100 %)	Urea-formaldehyde	13	1701	11.36	0.75	Face:	2 h:5.80	2 h:14.25 24 h:37.26	714	[37]
							1095	24 h:11.60			
							Top:				
							1006				
15	Pinus particles particleboards	Urea-formaldehyde	13	1815	12.94	0.60	Face:	2 h:5.73	2 h:14.97 24 h:34.75	682	[37]
							1067				
							Top:	24 h:14.30			
							1043				
16	Agricultural residues particleboards (Eucalyptus)	Urea-formaldehyde	15	2823	16.70	1		2 h:9.5	2 h:28.2	657	[48]
								24 h	24 h:37.8		
								14.9			
17	Agricultural residues particleboards (Pinus)	Urea-formaldehyde	15	2621	15.20	0.84		2 h:9.1	2 h:21.7	654	[48]
								24 h:15.6	24 h:39.0		
18	Agricultural residues particleboards (Sugarcane bagasse)	Urea-formaldehyde	15	2095	12.50	0.46		2 h:4.5	2 h:15.7	672	[48]
								24 h:17.8	24 h:47.1		
19	Agricultural residues particleboards (50 % eucalyptus+50 % bagasse)	Urea-formaldehyde	15	2567	15.70	0.92		2 h:7.4	2 h:18.0	659	[48]
								24 h:15.5	24 h:42.5		
20	Agricultural residues particleboards (50 % pinus+50 % bagasse)	Urea-formaldehyde	15	2480	14.50	0.63		2 h:5.5	2 h:16.8	648	[48]
								24 h:15.4	24 h:43.2		
21	Recycled paper boards (Cardboard)	Polylactic acid	18	2231.47 (Maximum)	10.82 (Maximum)	13.53 (Maximum)	Face:	2 h:0.43	2 h:4.18	632.25–837.75	[40]
							581.44 (Maximum)	(Minimum)	(Minimum)		
							Edge:	24 h:1.78	24 h:11.13		
							366.04 (Maximum)	(Minimum)	(Minimum)		
22	Recycled paper boards (Office paper)	Polylactic acid	18	1992.47 (Maximum)	6.57 (Maximum)	7.70 (Maximum)	Face:	2 h:3.54	2 h:27.54	655.74–874.69	[40]
							588.96 (Maximum)	(Minimum)	(Minimum)		
							Edge:	24 h:5.11	24 h:47.35		
							490.04 (Maximum)	(Minimum)	(Minimum)		
23	Recycled paper boards (Deinked pulp)	Urea-formaldehyde	20	1189.07	6.13	0.39	Face:	24 h:29.50 %		841.96	[43]
							1899.97				
24	Recycled paper boards (Old corrugated cardboard)	Urea-formaldehyde	20	750.83	5.89	0.51	Face:	24 h:21.03		659.10	[43]
							1277.94				

ID, identifier; THK, thickness; MOE, modulus of elasticity; MOR, modulus of rupture; IB, internal bonding; SWR, screw withdrawal resistance; TS, thickness swelling; WA, water absorption; D, density; REF, references.

The formulation of the standards ensures the product quality, performance, safety, and market development. The requirements specified in the standards are the specification limits for the product used to determine whether a unit product is qualified. In this study, recycled boards made from sustainable materials are planned for use in furniture production. Therefore, it is necessary to compare the relevant standards of recycled boards with the board standards formulated for furniture use.

A P2 particleboard is defined as a board used for interior decoration (including furniture) under dry conditions [49,50]. EN 312 is the European standard for particleboards, and is primarily used in European countries [49]. GB/T 4897 is the national standard issued by China for particleboards, and is mainly used in China [50]. Both standards contain relevant requirements for P2 particleboard. Therefore, benchmarking P2 particleboard in the EN 312 and GB/T 4897 standards can effectively test the application potential of boards made of sustainable furniture materials.

Both in the EN 312 and GB/T 4897 standards, corresponding data standards were set for different board thicknesses [49,50]. Therefore, to facilitate the comparison of the data in Table 1 with the data in subsequent standards, the order of the boards in Table 1 was determined according to their thickness (from thin to thick).

According to the EN 312 standard, the board thickness is divided into nine categories: <3 mm, 3–4 mm, > 4–6 mm, > 6–13 mm, >13–20 mm, >20–25 mm, >25–32 mm, >32–40 mm, and >40 mm [49]. In the GB/T 4897 standard, the thickness of the boards is divided into six categories: ≤6 mm, >6 mm–13 mm, >13 mm–20 mm, >20 mm–25 mm, >25 mm–34 mm, >34 mm [50].

Flexural properties are crucial for board materials used in furniture such as tabletops and countertops. As the board materials for furniture must be resistant to bending, the material should not shift when heavy objects are placed on the surface, and the modulus of elasticity (MOE) and modulus of rupture (MOR) are the two related indicators. Further, internal bonding (IB) is a very important property of wood-based products that have strong joints between each other in furniture made from board materials, such as particleboard [40]. Therefore, MOE, MOR, and IB are three important indicators for evaluating board performance.

In both standards, the thickness ranges that match the statistical range of this study are >6 mm–13 mm and >13 mm–20 mm. The MOE, MOR, and IB data requirements in EN 312 and GB/T 4897 standards were consistent. That is, MOE for boards >6 mm–13 mm: 1800 N/mm^2^, MOR:11.0 N/mm^2^, IB:0.40 N/mm^2^; MOE for boards >13 mm–20 mm: 1600 N/mm^2^, MOR:11.0 N/mm^2^, IB:0.35 N/mm^2^. Additionally, although the thickest plate size range in EN 312 (>40 mm) and the thickest plate size range in the GB/T 4897 standard (>34 mm) were inconsistent, the data requirements were consistent (MOE: 1050 N/mm^2^; MOR: 7.0 N/mm^2^, IB: 0.20 N/mm^2^) [49,50].

**Fig. 1:**
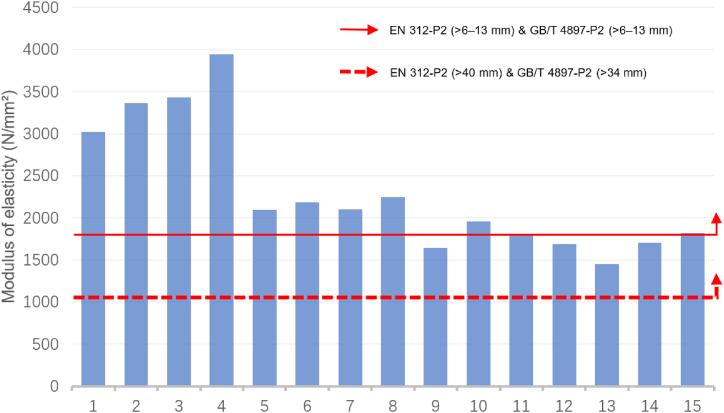
Comparison of the modulus of elasticity for identifiers 1 to 15 with the standards of EN 312-P2 boards & GB/T 4897-P2 boards.

Source: Authors, 2024

**Fig. 2:**
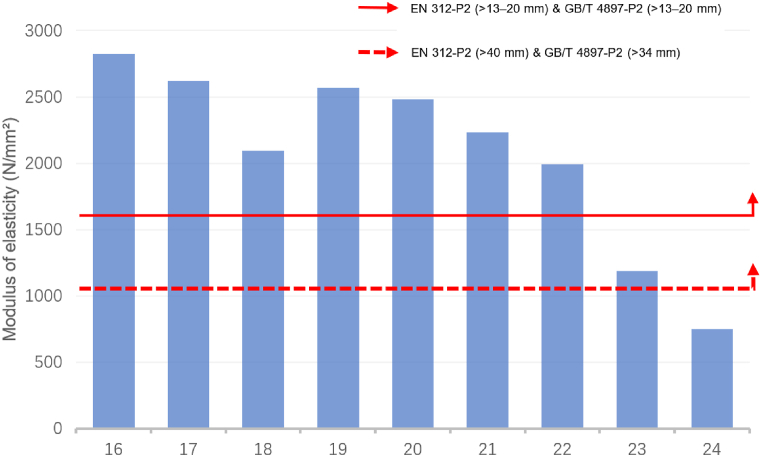
Comparison of the modulus of elasticity for identifiers 16 to 24 with the standards of EN 312-P2 boards & GB/T 4897-P2 boards.

Source: Authors, 2024

Fig. 1 and Fig. 2 exhibit a comparison of the MOE data of sustainable recycled boards with the EN 312 and GB/T 4897 standards. Fig. 1 illustrates a comparison of the MOE data of boards with thicknesses > 6–13 mm (ID: 1–15). Except for IDs 9, 12, 13, and 14, all boards met the standards. Fig. 2 displays a comparison of the MOE data of boards with thicknesses >13–20 mm (ID: 16–24). Except for IDs 23, and 24, all other boards met the standards.

For boards that do not meet these standards, various methods can be used to improve their performance, such as optimising material formulation, improving the production process, and surface treatment. Increasing the thickness of a material usually improves its MOE. Comparing the data standards for board thickness >40 mm in EN 312 and > 34 mm in GB/T 4897, it can be inferred that the performance of most sustainable recycled boards meets the requirements of the EN 312 and GB/T 4897 standards.

**Fig. 3:**
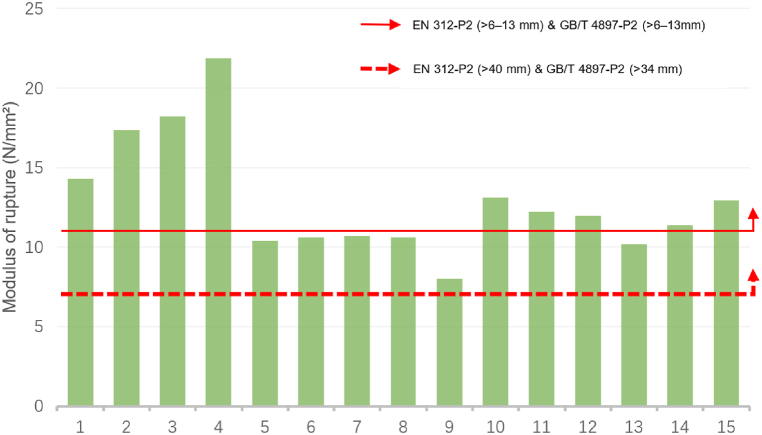
Comparison of the modulus of rupture for identifiers 1 to 15 with the standards of EN 312-P2 boards & GB/T 4897-P2 boards.

Source: Authors, 2024

**Fig. 4:**
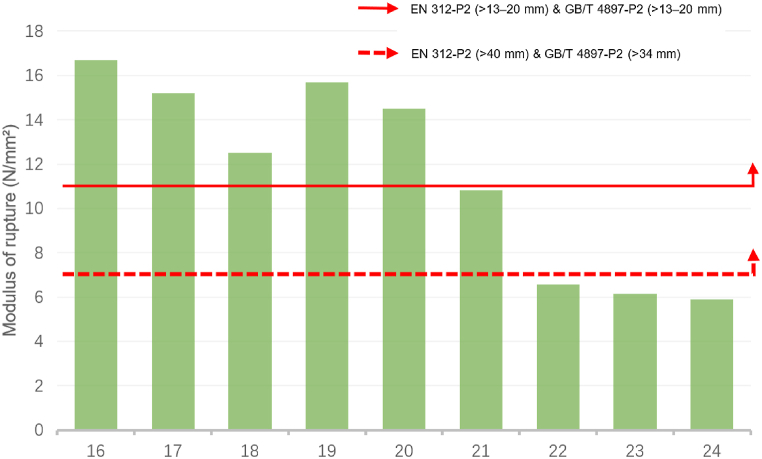
Comparison of the modulus of rupture for identifiers 16 to 24 with the standards of EN 312-P2 boards & GB/T 4897-P2 boards.

Source: Authors, 2024

Fig. 3 and Fig. 4 exhibit a comparison of the MOR data of sustainable recycled boards with the EN 312 and GB/T 4897 standards. Fig. 3 demonstrates the MOR data comparison of boards with thicknesses > 6–13 mm (ID: 1–15). Except for IDs 5, 6, 7, 8, 9, and 13, all boards met these standards. Fig. 4 displays the MOR data comparison of boards with thicknesses >13–20 mm (ID: 16–24). Except for IDs 21, 22, 23, and 24, all boards met the standards. Similarly, for boards that do not meet the standards, there are many methods to ensure that their performance meets the standards, such as material formulation optimisation, improved production processes, and surface treatment. Increasing the material thickness usually improves the MOR performance. By comparing the data standards for board thickness >40 mm in EN 312 and > 34 mm in GB/T 4897, it can be inferred that the performance of most sustainable recycled boards meets the MOR requirements of the EN 312 and GB/T 4897 standards.

**Fig. 5:**
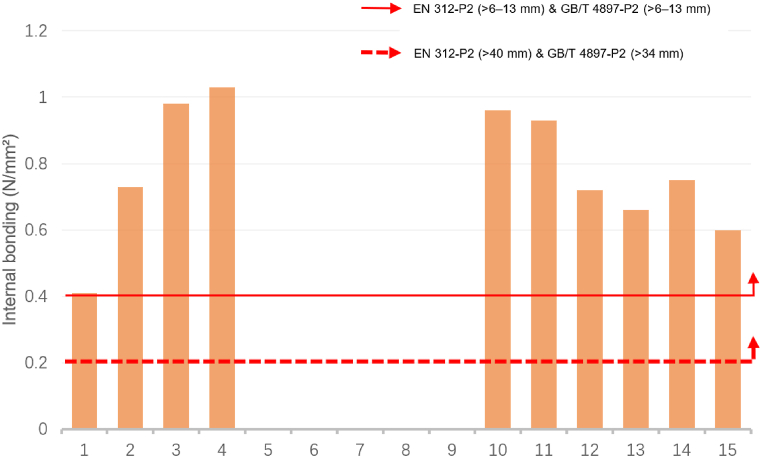
Comparison of the internal bonding for identifiers 1 to 15 with the standards of EN 312-P2 boards & GB/T 4897-P2 boards.

Source: Authors, 2024

**Fig. 6:**
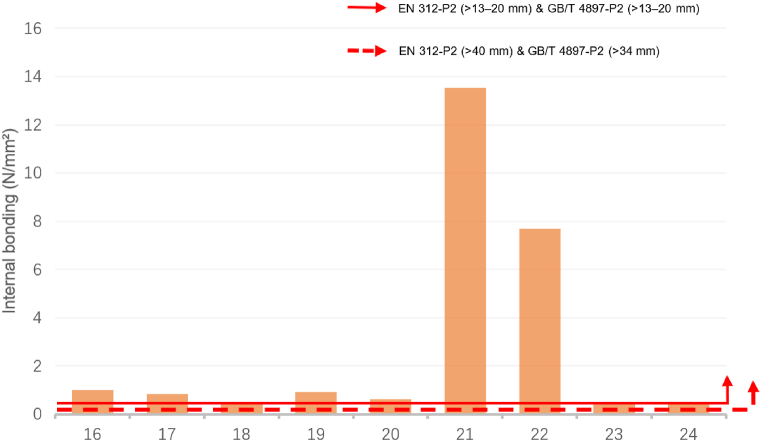
Comparison of the internal bonding for identifiers 16 to 24 with the standards of EN 312-P2 boards & GB/T 4897-P2 boards.

Source: Authors, 2024

Fig. 5 and Fig. 6 illustrate a comparison of the IB data of sustainable recycled panels with the EN 312 and GB/T 4897 standards. Fig. 5 exhibits the IB data comparison of panels with thicknesses > 6–13 mm (ID: 1–15). Except for IDs 5, 6, 7, 8, and 9, which have not been tested accordingly, all other panels meet the standards. Fig. 6 depicts the IB data comparison of panels with thicknesses >13–20 mm (ID: 16–24). All the panels meet the standards. Similarly, by comparing the data standards for board thickness >40 mm in EN 312 and > 34 mm in GB/T 4897, it can be inferred that the performance of sustainable recycled boards in the existing data can satisfy the requirements of IB in the EN 312 and GB/T 4897 standards.

In general, most boards made from sustainable materials satisfy all the requirements of the MOE, MOR, and IB in EN 312 and GB/T 4897. Therefore, sustainable materials (e.g. agricultural biomass, recycled wood, and recycled paper) are potential raw materials for boards used in furniture manufacturing.

## 4 Discussion

This study analyses and summarises the relevant literature on sustainable furniture, furniture rental, and sustainable material applications. Additionally, it aimed to assess the benefits of integrating various perspectives and provide recommendations for further research.

**Fig. 7:**
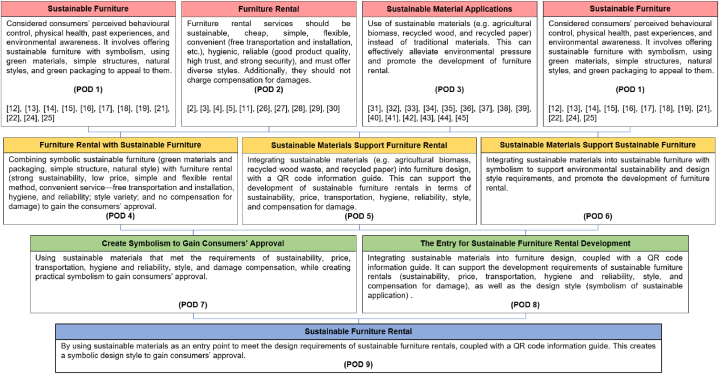
Theoretical development through sustainable furniture, furniture rental, and sustainable material applications.

Source: Authors, 2024

A comprehensive literature survey is exhibited in Fig. 7, and the results provide feasible hypotheses to answer the research questions. This study offers a final POD tree diagram [7] to support the comprehensive processes of sustainable furniture, furniture rental, and sustainable material applications. This section explains the process of combining preliminary findings to create final theoretical recommendations and suggests integration possibilities for future research.

Upon reviewing the results of the initial discussion, the researchers proposed POD1, which considered consumers’ perceived behavioural control, physical health, past experiences, and environmental awareness. It involves offering sustainable furniture with symbolism, using green materials, simple structures, natural styles, and green packaging to appeal to them. Next, based on the discussion results of the second theme, the researchers proposed POD2. They suggested that the furniture rental services should be sustainable, cheap, simple, flexible, convenient (free transportation and installation, etc.), hygienic, reliable (good product quality, high trust, and strong security), and must offer diverse styles. Additionally, they should not charge compensation for damages. Finally, based on the discussion of the third theme, the researchers proposed POD3, which suggested the use of sustainable materials (e.g. agricultural biomass, recycled wood, and recycled paper) instead of traditional materials. This can effectively alleviate environmental pressure and promote the development of furniture rental.

**Fig. 8:**
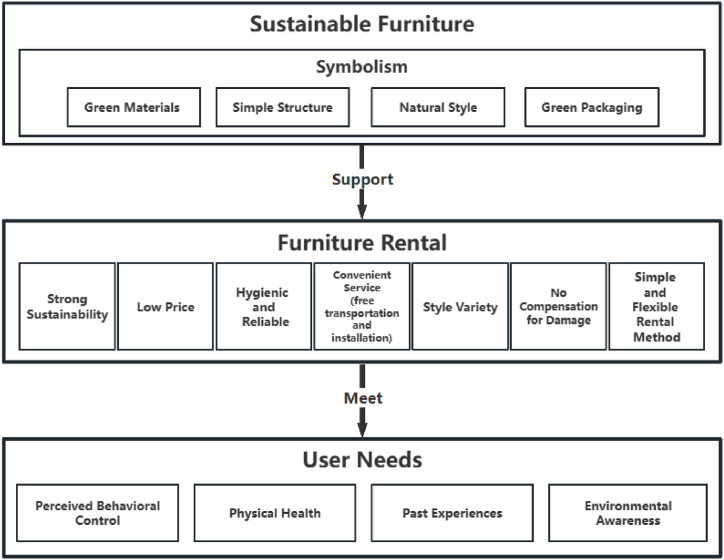
Support the concept of sustainable furniture and furniture rental integration.

Source: Authors, 2024

Further, by cross-synthesising POD1 and POD2, researchers observed that the benefits of sustainable furniture are closely aligned with those of furniture leasing in meeting consumer needs. Therefore, combining sustainable furniture with furniture leasing can provide better solutions to consumer needs. This resulted in POD4—combining symbolic sustainable furniture (green materials and packaging, simple structure, natural style) with furniture rental (strong sustainability, low price, simple and flexible rental method, convenient service—free transportation and installation, hygiene, and reliability; style variety; and no compensation for damage) to gain the consumers’ approval (Fig. 8).

Similarly, by combining POD2 and POD3, considering the inexpensive nature of rental furniture, using sustainable recycled materials instead of wood chips to produce boards for furniture manufacturing can significantly reduce furniture costs. Furthermore, respondents demand hygienic furniture that is not soiled or has pests and bugs from previous users. Moreover, furniture suppliers may not have fully disinfected the furniture. In addition, some respondents said they would not accept second-hand products because they have been used by other users. Considering the above mentioned factors, reliability—good product quality, high trust, strong security—combined with the recyclability of sustainable materials, the researchers proposed a new leasing model. In other words, when the first user ends the lease and returns the furniture to the leasing company, the leasing company chooses to recycle the furniture into raw materials to produce new furniture. The advantage of doing this is that it maximises the satisfaction of the user's hygiene requirements for furniture, while also improving the product's reliability as new furniture has to pass through a qualified inspection. Moreover, to reduce wastage, some furniture will be separately discarded (e.g. very short-term rentals with almost no damage or contamination to the furniture). Rental companies consider cleaning, disinfecting, and refurbishing this furniture and providing it to users who are more cost sensitive. This reiterates the economic requirements of rental furniture. Finally, with regard to the exemption from compensation for damage, in most cases, when users return rental furniture to the company, it will usually recycle it into materials for making new furniture instead of continuing to rent the furniture to other users. Therefore, this method can support user requirements for exemption from compensation for damages to rental furniture. Generally, the use of sustainable materials in furniture rentals can promote its development.

Additionally, with the rapid development of informatisation, information acquisition has become crucial. Efficient and transparent information transmission improves user experience and significantly increases market transparency and trust. Especially in the field of rental furniture, modern information technology such as QR codes can achieve rapid transmission and transparent displays of information. The advantage of using QR codes is that they can be scanned by mobile devices such as smartphones. Therefore, most people can easily access them [51]. This reiterates the past experiences of users. By scanning the QR code, users can easily obtain detailed information about the furniture, which includes the usage guide of the rental furniture, the quality standards that it meets, its lifecycle, detailed information on the materials used (material composition, material lifecycle, etc.), the properties of the furniture (new or refurbished, whether the refurbished furniture has been cleaned and disinfected, the degree of refurbishment, etc.), and factory inspection records (certificate of conformity and information on relevant responsible persons). The application of QR codes improves information transparency and enhances users’ trust and satisfaction with the rental furniture. Furthermore, it facilitates rental companies in making decisions after taking furniture back from users [51]. This resulted in POD5—integrating sustainable materials (e.g. agricultural biomass, recycled wood waste, and recycled paper) into furniture design, with a QR code information guide. This can support the development of sustainable furniture rentals in terms of sustainability, price, transportation, hygiene, reliability, style, and compensation for damage.

Furthermore, there is no unified quality standard for rental furniture. Therefore, the formulation of rental furniture standards is of great significance to ensure the quality, performance and safety of rental furniture and promote its market. Therefore, researchers suggest that the formulation of rental furniture standards should refer to existing furniture standards such as EN 12520 [52]. The implementation of rental furniture standards will help provide users with reliable and safe furniture, ensure customer satisfaction and trust, and improve the reliability of furniture (product quality, safety, and trust levels).

Next, by combining POD3 and POD1, researchers observed that the application of sustainable materials could also provide the necessary support for sustainable furniture (such as meeting consumers' environmental awareness and the symbolism created by the natural style of sustainable materials), further promoting the development of sustainable furniture. This resulted in POD6—integrating sustainable materials into sustainable furniture with symbolism to support environmental sustainability and design style requirements, and promote the development of furniture rental.

Furthermore, by cross-integrating POD4 and POD5, researchers observed that the use of sustainable materials assists in creating the symbolism of furniture through multiple aspects of sustainability, which is crucial for consumer appeal. This resulted in POD7—using sustainable materials that met the requirements of sustainability, price, transportation, hygiene and reliability, style, and damage compensation, while creating practical symbolism to gain consumers' approval.

**Fig. 9:**
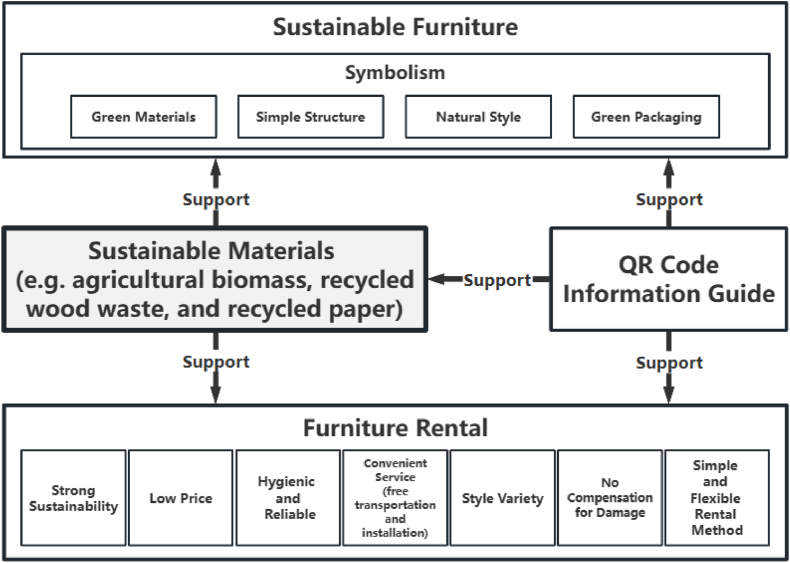
Sustainable materials (e.g. agricultural biomass, recycled wood waste, and recycled paper) support the concept of sustainable furniture and furniture rental.

Source: Authors, 2024

Next, by synthesising POD5 and POD6, the researchers observed that the application of sustainable materials can be used as an entry point for the development of sustainable furniture rental. This provides solutions to many problems encountered in the development process of sustainable furniture rental. This resulted in POD8—integrating sustainable materials into furniture design, coupled with a QR code information guide. It can support the development requirements of sustainable furniture rentals (sustainability, price, transportation, hygiene and reliability, style, and compensation for damage), as well as the design style (symbolism of sustainable application) (Fig. 9).

**Fig. 10:**
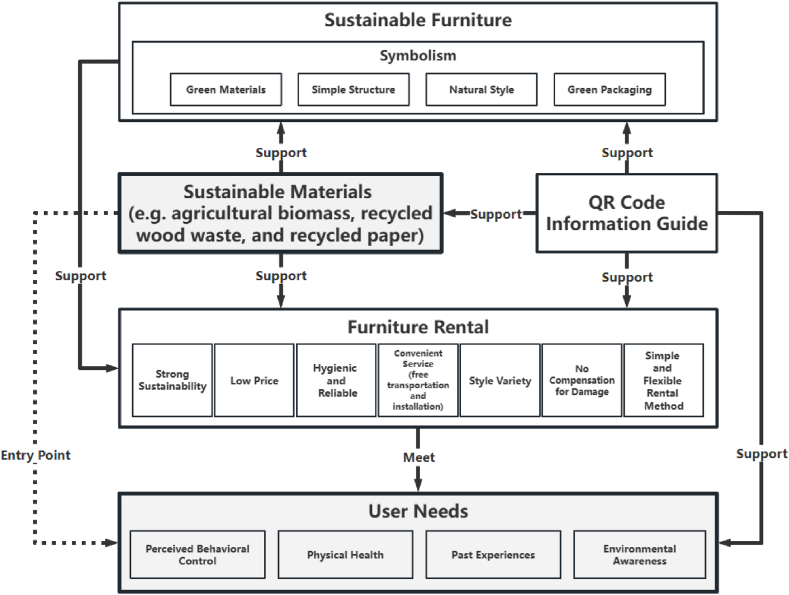
The construction of a sustainable furniture rental concept.

Source: Authors, 2024

Finally, POD7 and POD8 were combined to form POD9, by using sustainable materials as an entry point to meet the design requirements of sustainable furniture rentals, coupled with a QR code information guide. This creates a symbolic design style to gain consumers’ approval (Fig. 10).

## 5 Conclusions

In summary, designers can leverage the user-related knowledge discovered in this study as well as the design features of sustainable furniture and the requirements for furniture rental to create problem-solving products and penetrate the sustainable furniture rental trend. Consequently, consumers no longer need to worry about problems when changing their residences (such as furniture transportation, furniture damage, and compensation). This encourages consumers to engage in new ways of using furniture with excellent value, such as sustainable furniture rentals.

This study proposes that sustainable furniture rentals can adopt the characteristics of sustainable furniture, and include a QR code information guide. Additionally, they can provide symbolic sustainable furniture using green materials, simple structures, natural styles, and green packaging. It has complementary advantages and is sustainable, cheap, simple. Additionally, it offers flexible leasing methods, convenient services (free transportation and installation), is hygienic and reliable, and has diverse styles. Furthermore, furniture leasing that does not require compensation for damages. In addition, this solution can improve the furniture rental system, providing furniture usage experiences and better benefits to small apartment users. Therefore, integrating sustainable furniture into furniture rental and adding sustainable materials are effective ways to improve the furniture usage experience of small apartment users. Future research should focus on the three major themes of sustainable furniture, furniture rental, and sustainable material applications to determine theoretical propositions and their application to support the furniture needs of small-house consumers.

## CRediT authorship contribution statement

**Wei Liu:** Writing – review & editing, Writing – original draft, Visualization, Validation, Project administration, Methodology, Investigation, Formal analysis, Conceptualization. **Siti Mastura Md Ishak:** Supervision, Project administration. **Mohd Faiz Yahaya:** Supervision.

## Data availability statement

The data associated with the articles included in this review are available in the original sources.

## Declaration of competing interest

The authors declare that they have no known competing financial interests or personal relationships that could have appeared to influence the work reported in this paper.
